# Supplementary material for: Role of Tyrosine Kinase Syk in Thrombus Stabilisation at High Shear
Source: Int J Mol Sci. 2022 Jan 1;23(1):493. doi: 10.3390/ijms23010493 (PMC8745592; doi:10.3390/ijms23010493)
Supplement: Supplementary file 1 [file ijms-23-00493-s001.zip › ijms-1528948-supplementary.pdf]

Supplementary materials

Role of tyrosine kinase Syk in thrombus stabilisation at high shear

Gina Perrella,[1,2] Samantha J. Montague,[1] Helena C. Brown,[1,3] Lourdes Garcia Quintanilla,[1] Alexandre Slater,[1] David Stegner,[3] Mark Thomas,[1] Johan W.M. Heemskerk,[2,4] Steve P. Watson[1,5]

[1]Institute of Cardiovascular Sciences, College of Medical and Dental Sciences, University of Birmingham, Edgbaston, Birmingham B15 2TT, UK; [2]Dept. of Biochemistry, CARIM, Maastricht University, Maastricht, NL; [3]Institute of Experimental Biomedicine I, University Hospital, University of Würzburg, Würzburg, DE; [4]Synapse Research Institute, 6214 AC Maastricht, NL; [5]COMPARE, The Universities of Birmingham and Nottingham, The Midlands, UK.

Materials and Methods

Antibodies

Target antigen	Vendor or Source	Working concentrations
HRP-conjugated sheep α-mouse and donkey α-rabbit IgG	GE Healthcare (Little Chalfont, UK)	1:10000
phospho-specific pAb against LAT (pY200)	Abcam (Cambridge, UK)	1:500
anti-Syk mAb (4D10)	Cambridge Bioscience (Cambridge, UK)	1:200
phospholipase C (PLC)γ2 mAb (Q-20)	Cambridge Bioscience (Cambridge, UK)	1:200
anti-P-tyrosine mAb (4G10)	Cambridge Bioscience (Cambridge, UK)	1:1000
phycoerythrin (PE)-labelled mouse anti-human platelet GPVI mAb (clone HY101)	BD Pharmingen (San Diego, CA, USA)	1:200
APC-labelled mouse anti-human CD41a mAb (clone HIP8)	BD Pharmingen (San Diego, CA, USA)	1:100

fluorescein isothiocyanate (FITC)-labelled polyclonal rabbit anti-human fibrinogen	Dako (Santa Clara, CA, USA)	1:100

Other reagents

Target antigen	Vendor or Source
D-Phe-Pro-Arg chloromethyl ketone (PPACK)	Cambridge Bioscience (Cambridge, UK)
protein Sepharose A, bovine serum albumin (BSA, fatty acid free, ≥96%)	Thermo Fisher Scientific (Waltham, MA, USA)
NuPAGETM 4-12% Bis-Tris gel from	Thermo Fisher Scientific (Waltham, MA, USA)
PRT-060318	Caltag Medsystems (Buckingham, UK)
Ibrutinib	Toronto Research Chemicals (North York, ON, Canada)
Dasatinib	LC Laboratories (Woburn, MA, USA)
Ticagrelor	Sigma-Aldrich (Zwijndrecht, NL)
Thrombin	Sigma-Aldrich (Zwijndrecht, NL)
Indomethacin	AstraZeneca (The Hague, NL)
Eptifibatide	GSK (Brentford, UK)
Horm collagen	Nicomed (Munich, Germany)
Collagen diluent	Nicomed (Munich, Germany)
Thrombin-activating peptide (TRAP, SFLLNR)	Alta Bioscience (Redditch, UK)
Fibrinogen	Enzyme Research (Swansea, UK)
Heparin	Wockhardt (Wrexham, UK)
